# Supplementary material for: Patterns of Intron Gain and Loss in Fungi
Source: PLoS Biol. 2004 Nov 30;2(12):e422. doi: 10.1371/journal.pbio.0020422 (PMC532390; doi:10.1371/journal.pbio.0020422)
Supplement: Table S1 — Also available at http://genes.mit.edu/NielsenEtAl/. (4.3 MB ZIP). [file pbio.0020422.st001.zip › NielsenEtAl/html/484.html]

AN1367.1.NCU03321.1.MG09313.1.FG11603.1


```
 CLUSTAL W (1.82) Multiple Sequence Alignments - Introns Inserted


Sequence 1: NCU03321.1	779 aa
Sequence 2: FG11603.1	765 aa
Sequence 3: MG09313.1	777 aa
Sequence 4: AN1367.1	799 aa
Alignment Length: 817 aa
Number Identitical Residues: 364 aa
Alignment Score (without introns) 19330


MG09313.1 	---~---MAPTAPAKKRKQS-----AEVTELSDDEIIDGLLDGALSQSED--DSDFVASG
NCU03321.1	---~---MGPKATE-KKRKV-----QDAEPESDDEIA-PDFDGALSQSEDEFDSDFDDN-
FG11603.1 	---~---MAPTTLPSRKRKV-----LAEPAPVDDELAGVELDGILSQSED--DSDFE---
AN1367.1  	MEL0LPPMAALKASKKRKAVTRDVEEEAGVFSGDELSSKDLDGALSDNAHDLLSDEDASD
          	 .   ..*..    .:::  : . .       .**: .  :** **:. ..  **   ..

MG09313.1 	DEDEEDEDEDEDEDKD---------EDDEHDDKQDIGDLQDLSLNDLDDDAQSKELAKDG
NCU03321.1	EHEQDGEQESGSEASDDEDEEGSLLSDDIPSDAEGDKAMGKLVLEDEEEELE------IE
FG11603.1 	ESDVEGELHDNSSEND---------LDEHDQDAEGDDDDDDAYSEDASSDAG------DS
AN1367.1  	SEIELIDDYSSSEEED---------DEELDSDEIPSEGEDEGVVKKKSTAVLD----LDG
          	.     :  . .. .*          ::  .*        .   :. .    .       

MG09313.1 	ANGGSGDGPEADDEDRPNYRVVEDANGGIRYVYD~EINPVYDSDDSDKEED-NRIGDIPL
NCU03321.1	IPGVDPKRPEKDDGD-KNYRIEKDANGNERYVYD2EIDPVYDSDDSDAQEAPNTIGNIPM
FG11603.1 	FG--APPTLDADNSE-PNYRVVKDANGGERYEYA~EVDPVYDSDDSDAQGPVNTIGDIPL
AN1367.1  	LEDREESTKEEEEEEQLNYRIEKDANGNDRYVYD~EINPDDNSDYSDVDENSNTIGNIPL
          	  .      : :: :  ***: :****. ** *  *::*  :** ** :   * **:**:

MG09313.1 	SFYEAYPHVGYTIDGKKLMRPSERRQALDSLLDSIEIPKGWTGLTDPTTGKPLNLS~QDE
NCU03321.1	SFYDSYPHIGYDINGKKIMRPATG-DALDALLDSIEVPKGWTGLTDIHTGNPLNLN2DDE
FG11603.1 	SFYDSYPHIGYDINGKKIMRPATG-EALDALLDSIEIPKGWTGLTDPETGKPLDLT~QDQ
AN1367.1  	SFYDQYPHIGYDINGKKILRPAKG-EALDALLDSIEIPKGWTGLTDPSTGKPLELS~QDE
          	***: ***:** *:***::**:   :***:******:*********  **:**:*. :*:

MG09313.1 	LELLKRVQMNEIPEEGYDPYP0DTVEYFTGIEEKMP~LSAAPEPKRRFIPSKHEAKRVMK
NCU03321.1	LELVRRVQMGMIPNELENPYP~ETVEYFTGIEEKMP1LSAAPEPKRRFLPSKNEAKKIMK
FG11603.1 	LELLKRLQMNEVPNEGYDPYP0DMVPYFTNIEEKMP~LSAAPEPKRRFVPSKHEAKRVAK
AN1367.1  	LELLRKVQMNEIPDEEYNPYE~PLTEWFTTEKEIMP~LSAAPEPKRRFVPSKHEAKRVMK
          	***::::**. :*:*  :**    . :**  :* ** ***********:***:***:: *

MG09313.1 	LVRAIREGRIQPYKSKEEREKEEEGKEEKYYDVWQDEQPRDPHVMHIPAPKLAPPGYDMS
NCU03321.1	LVRAIREGRILPYKPPEEREKEEEE-EEQYFDIWQDEVPQEVNPLHIPAPKLPPPGFDMS
FG11603.1 	LVRAIKEGRILPYKPPEERQREDEEKEESYYDVWANEEPQDPHVMNIPAPKLAPPGYDLS
AN1367.1  	IVKAIREGRILPFKP--QTQEDEEDKDVIKYDLWADEAERPDHPMHVPAPKLPPPGYEES
          	:*:**:**** *:*.  : :.::* .:   :*:* :*  :  : :::*****.***:: *

MG09313.1 	YNPPPEYLPTKAEREEWEKMDPEDREKEYLPQKFDALRKVPGYETFVKERFERCLDLYLA
NCU03321.1	YNPPAEYLPTKEEREEWEKLDPEEREKEYLPQKYDSLRKVPGYGEIVKERFERCMDLYLA
FG11603.1 	YNPPPEYLPTEEEKEAWKNQDPEEREKEYLPAKFDSLRKVPGYGEFVKERFDRCLDLYLA
AN1367.1  	YHPPPEYLPDKKERKAWEDADPEDREREFLPQDFGSLRRVPGYENFVKEKFERCLDLYLA
          	*:**.**** : *:: *:. ***:**:*:** .:.:**:****  :***:*:**:*****

MG09313.1 	PRVRKNRLNIDPASLLPKLPRPEELKPFPTVCQAIFRGHEGRVRSSAIDPTGLWLATGGD
NCU03321.1	PRVRKNRLNIDPNSLLPKLPSPSELKPFPTVAQTVFRGHDGRVRSVAIDPTGVAVASGGD
FG11603.1 	PRIRKNRLNIDPNSLLPKLPRPEDLKPFPTLNQTIFRGHDGRVRSVAFSPDGEFVASGGD
AN1367.1  	PRVRRSKLNIDPESLLPKLPSPEELKPFPTACAAVFRGHKGRVRSLAVDPTGIWLATGGD
          	**:*:.:***** ******* *.:******   ::****.***** *..* *  :*:***

MG09313.1 	DGYVRIWLINPARQVWAVKLSSDEAVNAVRWRPTKDTMILAAAAGEEIFLMVPPDID--P
NCU03321.1	DGTVRVWELLTGRQVWSVKLSSEEAVNTVRWRPAKDSFILSAAVGEDVYLMVPTHASVTP
FG11603.1 	DGTVRVWALN-GHQEWMAKLNSEEPVNMVRWRPNKETFILAAAAGEDLFFMIPSVAS--D
AN1367.1  	DGTVRVWELITGRQLWSVKLGEDEPVNVVRWRPGKDAVILAAAAGDDIFLAVPPIAD--P
          	** **:* : ..:* * .**..:*.** ***** *::.**:**.*::::: :*.  .   

MG09313.1 	EVEQTSRAVLDAGFGHAAGGADKQTTDG--KAPAAKWARPGARLEDEGVLVKVTVRSPVK
NCU03321.1	ALDQASRDILSAGFGYATNGQQHGAAPG--KEPPAKWARPGAKLEDEGVLIRITVRSTVK
FG11603.1 	AVEKASREVLDVGFGYATNGAQSSAMNGIKKEPPAKWSRPGAKLEAAGVLLMATVRSAIK
AN1367.1  	ETEKASLDLLDAGWGYAASKPAPKPADEEKKSTPPQWIRPSAALADSGVCVVIPLRYIAK
          	  :::*  :*..*:*:*:.     .    .* ...:* **.* *   ** :  .:*   *

MG09313.1 	VISWHRRGDHFCTVSP---SGQRSSVAVHTLSKHLSQIPFR---KLSGLAQVAHFHPSRP
NCU03321.1	VINWHRKGDHFATVSP---SGQRSSVAIHTLSKHLTQIPFR---KLSGLAQTASFHPLRP
FG11603.1 	VINWHKRGEYICTVSP---TGQRSSVAIHAISKHLSQLPFR---KLPGLAQTAQFHPSRP
AN1367.1  	TISWHRRGDYFVTVCSGASTPASVAIAIHTVSKHLTQYPFRRRIKGGGPPQTAHFHPSKP
          	.*.**::*::: **...:::    ::*:*::****:* ***   *  * .*.* *** :*

MG09313.1 	LFFVATQRTIRCYDLQRLELVKVVQPGARW~ISSFDIHP------GGDNLIVGSYDRRLL
NCU03321.1	LFFVATQRTIRCYDLQKLELVKVVQPGAKW2ISSFDIHP------GGDNLIVGSYDKRLL
FG11603.1 	LFFVATQRMIRCYDLQREELVKVVQPGARW~ISSFDVHP------GGDNLIVGSYDRRLL
AN1367.1  	ILFVANQRTIRAYDLSRQLLVKIVQPGARW~ISSFDIHPTSATAAGGDNLIVGSYDRRLL
          	::***.** **.***.:  ***:*****:* *****:**::::::***********:***

MG09313.1 	WHDLDLSTRPYKTMRFHPQAIRAVKYHRG-GLPLFADASDDGSLQIFHGKVVSDLMENAT
NCU03321.1	WHDLDLSIRPYKTMRFHSEAVRQVKYHRG-GLPLFADASDDGTLQIFHGKVPNDQLENPT
FG11603.1 	WHDLDLSARPYKTMRFHPEAIRAVKFHR--TLPLFADASDDGTLQIFHGKVVSDLMENAT
AN1367.1  	WHDLELSQRPYKTLRYHRKAIRAVKFHPGGRYPLFADASDDGSLQIFHGNVTGDMLSNAT
          	****:** *****:*:* :*:* **:* ..  **********:******:* .* :.*.*

MG09313.1 	IVPLKSLKGHRVVDSLGVMDVDWHPSEPWCISAGADGTCRLWM
NCU03321.1	IVPVKVLKGHKVVNKLGVLDMDWHPREPWCVSAGADGTIRLWM
FG11603.1 	IVPVKMLRGHKVISKLGVLDVDWHPKHPWCLSAGADGTCRLWT
AN1367.1  	IVPLKVLRGHKITGELGVLDIDWHPREAWCVSAGADGTCRLWM
          	***:* *:**:: ..***:*:**** ..**:******* ***
```
